# Does integrated care reduce hospital activity for patients with chronic diseases? An umbrella review of systematic reviews

**DOI:** 10.1136/bmjopen-2016-011952

**Published:** 2016-11-21

**Authors:** Sarah Damery, Sarah Flanagan, Gill Combes

**Affiliations:** Institute of Applied Health Research, College of Medical and Dental Sciences, University of Birmingham, Edgbaston, UK

**Keywords:** umbrella review, integrated care, chronic disease, review of reviews, resource use, hospital activity

## Abstract

**Objective:**

To summarise the evidence regarding the effectiveness of integrated care interventions in reducing hospital activity.

**Design:**

Umbrella review of systematic reviews and meta-analyses.

**Setting:**

Interventions must have delivered care crossing the boundary between at least two health and/or social care settings.

**Participants:**

Adult patients with one or more chronic diseases.

**Data sources:**

MEDLINE, Embase, ASSIA, PsycINFO, HMIC, CINAHL, Cochrane Library (HTA database, DARE, Cochrane Database of Systematic Reviews), EPPI-Centre, TRIP, HEED, manual screening of references.

**Outcome measures:**

Any measure of hospital admission or readmission, length of stay (LoS), accident and emergency use, healthcare costs.

**Results:**

50 reviews were included. Interventions focused on case management (n=8), chronic care model (CCM) (n=9), discharge management (n=15), complex interventions (n=3), multidisciplinary teams (MDT) (n=10) and self-management (n=5). 29 reviews reported statistically significant improvements in at least one outcome. 11/21 reviews reported significantly reduced emergency admissions (15–50%); 11/24 showed significant reductions in all-cause (10–30%) or condition-specific (15–50%) readmissions; 9/16 reported LoS reductions of 1–7 days and 4/9 showed significantly lower A&E use (30–40%). 10/25 reviews reported significant cost reductions but provided little robust evidence. Effective interventions included discharge management with postdischarge support, MDT care with teams that include condition-specific expertise, specialist nurses and/or pharmacists and self-management as an adjunct to broader interventions. Interventions were most effective when targeting single conditions such as heart failure, and when care was provided in patients’ homes.

**Conclusions:**

Although all outcomes showed some significant reductions, and a number of potentially effective interventions were found, interventions rarely demonstrated unequivocally positive effects. Despite the centrality of integrated care to current policy, questions remain about whether the magnitude of potentially achievable gains is enough to satisfy national targets for reductions in hospital activity.

**Trial registration number:**

CRD42015016458.

**Strengths and limitations of this study**

- This umbrella review is the first of its kind since integrated care became central to healthcare policy.
- Outcomes were selected following consultation with service providers, commissioners and patient representatives to ensure relevance.
- We assessed a large volume of international evidence across diverse chronic conditions, interventions and outcomes.
- Umbrella reviews do not allow conclusions to be drawn about the detailed contexts in which interventions were implemented, but they do permit a broader overview of the evidence base than would be possible with a focus on primary research alone.
- Heterogeneity of intervention design, duration, intensity and follow-up prohibited meta-synthesis across reviews.

## Introduction

Hospital activity continues to rise and currently accounts for almost half of annual NHS expenditure.^1^ Demands on the acute sector are strongly influenced by the rapidly growing number of patients with multiple, chronic health conditions. These patients often need to access multiple health and social care settings but typically experience fragmented and poorly coordinated care.^2^ ^3^ Reducing hospital activity is seen as the key to relieving pressure on services that are rapidly approaching their limits,^4^ and integrated care has become a cornerstone of the policy response to this challenge in the UK and most other developed countries. Integrated care represents an organising principle for care delivery that aims to improve patient experience of services through improved coordination across and between settings.^5^ By facilitating more patient contact, treatment and follow-up in primary care, in the community or in patients’ homes, integration aims to reduce substantially the number of emergency and other admissions to hospital and facilitate timely and effective discharge from hospital to other settings. Following the establishment of a series of integrated care ‘pioneers’ in 2013, hospital trusts and commissioning organisations in England are planning and investing in a plethora of integrated services via the Better Care Fund (BCF), which aims to promote joint working at a strategic and operational level.^6^ Following the NHS Five Year Forward View,^7^ there are also proposals to develop and implement new models of care with integration as their central principle.^8^

Integration undoubtedly has laudable aims—poor care coordination is often the main problem cited by patients when describing their experiences of health and social care services.^9^ ^10^ NHS staff also welcome integration,^11^ ^12^ yet evidence about the effectiveness of integrated care in reducing healthcare resource use, particularly within the acute sector, is limited. Integrated care programmes can have a positive effect on service quality,^13^ and there is emerging evidence from recent evaluations of integrated care pilots that suggests potential for service efficiencies.^14^ ^15^ However, there is still uncertainty about which interventions are most effective and how these should be implemented,^16^ alongside persistent questions over whether the aims of integration are ultimately achievable in any meaningful way.^17^ Given this uncertainty, it is timely to assess the evidence. This paper reports the findings of an umbrella review of the evidence for integrated care interventions operating across health and/or social care settings for chronic disease management in order to assess: (1) whether integration reduces hospital activity, (2) which interventions are the most promising, for which patients and in which settings, and (3) what are the associated cost implications.

## Methods

Umbrella reviews synthesise evidence from multiple systematic reviews into a single ‘meta review’, using the findings and conclusions of included systematic reviews as the raw data. They are useful when the evidence base is broad and are of particular importance for decision makers who need a synthesis of the most current and reliable data relevant to their context.^18^ The protocol was published^19^ and registered on PROSPERO.

### Inclusion criteria

We included systematic reviews and meta-analyses published since January 2000 that evaluated interventions designed to facilitate integrated health and/or social care services. The year 2000 was chosen following scoping searches that indicated little or no systematic review evidence for integrated care interventions before this date. Eligible reviews could include primary studies of any experimental or quasi-experimental study design, providing the authors had identified studies using systematic methods. Eligibility was limited to reviews available in English.

Participants included adult patients with one or more chronic conditions. A list of 11 specific conditions was derived following a scoping review and combined a series of conditions recommended as central to any systematic review of chronic disease^20^ ^21^ and those included in the most recent Health Survey for England.^22^ The resulting conditions (hypertension, depression, diabetes, coronary heart disease, stroke, transient ischaemic attack, chronic obstructive pulmonary disease (COPD), cancer, heart failure, dementia and arthritis) covered those that are most prevalent within the adult population, most costly to manage and most likely to occur in combination with other chronic conditions.

Interventions could be implemented in any health or social care setting (primary, secondary or community care), as long as they crossed the boundary between two or more settings. The community setting encompassed care given in the community, in patient homes or by social care professionals. Exclusion criteria were: palliative care interventions; purely psychosocial interventions or those related to spirituality, mindfulness, health literacy or the use of complementary and alternative medicines; interventions focusing solely on diet and lifestyle factors; treatment or medication adherence; the effectiveness of surgical or diagnostic techniques; caregivers; pregnancy, and interventions implemented in less economically developed countries.

Comparison groups could include usual care, no intervention or comparison to one or more other interventions.

### Outcome measures

Outcome measures were selected following a scoping review, a stakeholder workshop attended by service providers and commissioners and consultation with a group of patient and public involvement (PPI) advisors. Eligible reviews assessed one or more of the following outcomes: acute sector activity (emergency hospital admissions/readmissions, length of hospital stay, accident and emergency (A&E) use) and healthcare costs.

### Search strategy

The search strategy was intentionally broad and included general terms related to chronic disease, multimorbidities and long-term conditions as well as MeSH terms for the 11 specific chronic diseases identified from scoping searches. Search terms associated with integrated care and known interventions were also included. A separate search identified systematic reviews that assessed the cost implications of integrated care interventions (see online supplementary information for MEDLINE search strategy).

10.1136/bmjopen-2016-011952.supp1 supplementary data

Relevant reviews were identified by searching electronic bibliographic databases and the manual checking of each included review's reference list. We searched MEDLINE, Embase, ASSIA (Applied Social Sciences Index and Abstracts), PsycINFO, Health Management Information Consortium database (HMIC), CINAHL, Cochrane library (including the Health Technology Assessment (HTA) database, Cochrane Database of Systematic Reviews, Database of Abstracts of Reviews of Effectiveness—DARE), EPPI-Centre library, TRIP database and the Health Economics Evaluations Database (HEED). Searches were performed in July 2014 and updated in December 2015.

### Eligibility assessment and data extraction

Two authors (SD and SF) independently screened titles and abstracts against the inclusion and exclusion criteria, and full text copies of all potentially relevant reviews were assessed. Disagreements were resolved through the independent assessment of a third author (GC). Where multiple versions of an eligible review were available, the most recent or most comprehensive version was included. Where the same review was published more than once (eg, Cochrane Collaboration review and subsequent update), the updated version was included. Data on review characteristics (databases searched, geographical scope, healthcare settings and disease(s) focused on), methodology (aim, research questions, number of studies included, review type), study participants, interventions and outcomes of interest were extracted from each included review and cross-checked by SD and SF according to a predefined data extraction sheet. For narrative reviews, a statement summarising the authors’ primary interpretation of findings was extracted. For meta-analyses, data on relative risks or ORs were extracted along with the corresponding 95% CIs.

### Quality assessment

Review quality was appraised independently by SD and SF using the Centre for Evidence-Based Medicine (CEBM) tool for critical appraisal of systematic reviews (http://www.cebm.net/wp-content/uploads/2014/04/SR_Appraisal_sheet_2005_English.doc) which scores each review between 0 (poor quality) and 5 (high quality). Using quality score as an exclusion criterion was not part of the protocol, but considerable quality differences were evident between reviews scoring 0 to 2.5 and those scoring 3 or above. Lower quality reviews had little (if any) extractable outcomes data so we decided to exclude reviews scoring 2.5 or less on the quality scale.

### Data analysis

Heterogeneity in study populations, interventions assessed, follow-up periods and specification of control groups prevented pooling of intervention effects and quantitative meta-synthesis across reviews. Owing to this, and to avoid the risk of ‘double counting’ evidence where multiple reviews contained some of the same primary studies, our synthesis was a primarily narrative review of interventions and outcomes. The strength of evidence from each meta-analysis or narrative review was characterised according to four categories: definite positive or negative associations, mixed findings or no association (table 1).

**Table 1:** Categorisation of the strength of effect for included reviews

Category	Symbol	Interpretation
Positive association	+	At least half of a review's included primary studies showed a statistically significant *increase* in a particular outcome following the intervention; the authors’ summary of findings (narrative reviews) demonstrated a positive association, or pooled results from a meta-analysis indicated a statistically significant positive association, eg, hospital admission rates significantly *increased.*
Negative association	−	At least half of a review's included primary studies showed a statistically significant *decrease* in a particular outcome following the intervention; the authors’ summary of findings (narrative reviews) demonstrated a negative association, or pooled results from a meta-analysis indicated a statistically significant negative association, eg, hospital admission rates significantly *reduced.*
Mixed findings	?	A review reported mixed findings, in which some primary studies may have shown a statistically significant difference between intervention and control groups whereas other primary studies showed no significant differences between groups.
No association	=	A review where no significant differences between intervention and control groups were reported in any of the included primary studies.

## Results

**Figure 1:**
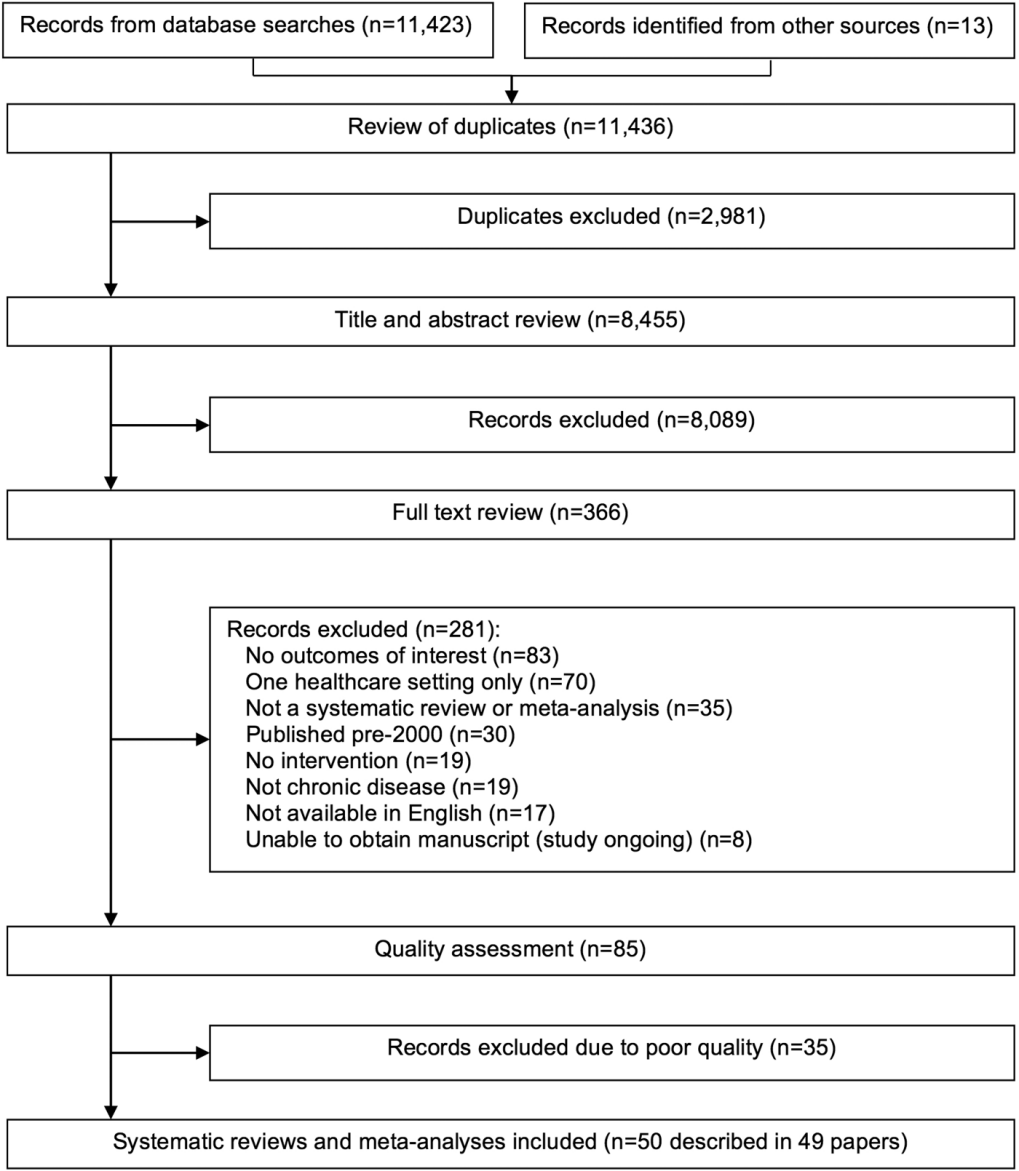
PRISMA diagram of search results.

Figure 1 summarises the search. A total of 11 436 potentially eligible reviews were identified, and 50 (in 49 papers) were included (table 2). A total of 1208 individual primary studies were included in the reviews (median 19, range 4–153). Nineteen reviews did not specify patient numbers, but across the 31 that did, all but three included 1000 or more patients (total 219 475, median 2692, range 857–78 590). Studies within reviews varied in duration from 2 weeks to 60 months, with most lasting up to 12 months, although 9 reviews did not specify follow-up duration. Furthermore, 21 reviews were narrative,^23–42^ 26 included meta-analyses^43–68^ and 3 were reviews of reviews.^69–71^

**Table 2:** Characteristics of included reviews

Author (year); country	Study types, n; *participants n*; databases	Condition(s); population(s); review type	Comparator; QA score; *time scale*	Intervention summary; health/social care settings	Findings of review by outcome (intervention vs control)
*Case management*					
Hickam *et al* (2013);^23^ USA	RCTs, observational n=153 *Not specified* 5 databases, inception—2011	Chronic disease Adult patients with complex care needs Narrative	Care without a case management component 4 *6–36 months*	Intensive interventions: multiple face-to-face interactions, home visits vs less intensive interventions: infrequent contact Delivered by case managers working alone or within a MDT of health professionals. Primary, secondary, community	*Admissions*: 2 studies found case management to be more effective in patients with greater disease burden. A further 4 studies found case management to be effective when case managers have greater levels of personal contact with patients (low quality evidence). *Costs* (4 studies): 3 found no difference between groups. One study found higher overall costs in intervention group vs control.
Hutt *et al* (2004);^24^ UK	RCTs, CCTs, before/after n=20 *n=18 002* ‘Major’ databases, 1996–2004	Chronic disease Over 65s with any chronic condition (mental health excluded) Narrative	Care without a case management component 3 *At least 3 months*	Home visits and/or periodic reassessment, ranging from case manager assessment at hospital or home with occasional telephone contact, to regular intensive contact where case managers arranged medical appointments and were contactable 24/7. Delivered by case manager (nurse or social worker). Primary, secondary, community.	*Admissions* (18 studies): 5 showed a significant reduction in admissions in intervention groups; 7 found no difference between groups; 4 found non-significant reductions, 2 found non-significant *increases* in admissions in the intervention group *LoS* (16 studies): 3/16 showed significant decrease, 2/16 showed non-significant increase, 11/16 showed no differences. *A&E use* (8 studies): 3 showed significant reduction, 2 showed significant increases, 2 showed non-significant increases. *Costs* (10 studies): 4 showed non-significant increases in intervention; 6 reported reductions although only 1 was significant.
Latour *et al* (2007);^25^ Netherlands	RCTs, CCTs, before/after n=10 *n=5092* 4 databases, inception-2005	Chronic disease Adult patients with acute or chronic conditions Narrative	Care without a case management component 3 *3–18 months*	Postdischarge nurse-led case management for complex patients, delivered in the outpatient setting. Needs assessment, service plans, monitoring, assessment, evaluation, follow-up via home visits and/or telephone. Secondary, community	*Readmissions* (9 studies): 3 high quality, 1 low quality reported positive results for intervention. 4 studies (2 high quality) showed no difference between groups; 1 presented insufficient data. *LoS* (6 studies): 2 showed significant reduction, 2 showed non-significant reduction, 2 showed no difference between groups. *A&E use* (4 studies): Strong evidence that intervention had no significant impact.
Manderson *et al* (2012);^26^ Canada	RCTs n=15 *n=2317* 5 databases, 1999–2011	Chronic disease Older people Narrative	Not specified 3 *1–18 months*	Care planning and coordination via phone support, home visits, liaison with medical and community services and/or education. APN, care coordinators, case managers. Primary, secondary, community	*Costs* (9 studies): 5 reported positive economic outcomes, 4 did not. Specific data and effect sizes not given.
Oeseburg *et al* (2009);^27^ Netherlands	RCTs n=9 *n=15 746* 3 databases, 1995–2007	Chronic disease Community dwelling patients Narrative	Care without a case management component 3 *10–36 months*	Home visits and/or telephone calls. Delivered by a case manager (nurse, social worker or nurse practitioner) who was either a member of a MDT or acted independently. Primary, secondary, community	*Admissions* (6 studies): 1 showed small reduction in favour of intervention (good quality). One found small increase in intervention group (weak quality). *LoS* (5 studies): One reported small reduction in days per year in hospital in intervention group. *A&E use* (5 studies): One reported small reduction in intervention, 1 reported an increase. 3 reported no difference. *Costs* (3 studies): 1 reported significant but trivial saving in intervention. Another found 19% cost reduction due to savings in nursing home, hospital and community costs. A third found costs to be higher in intervention (non-significant).
Stokes *et al* (2015);^43^ UK	RCTs, CCTs, before/after, time series n=36 *n=23 711* 6 databases, inception-2014	Chronic disease Adult patients with chronic diseases SR and meta-analysis	Care without a case management component 4 *6–60 months*	Community-based MDTs responsible for delivering and coordinating services; MDT care plan following case worker assessment, case manager constantly available to deal with problems. Delivered by care manager, nurse, pharmacist, GP collaborating with nurse. Primary, community	*Costs*: No significant effects found: Short term (0–12 months): SMD −0.00 CI −0/07 to 0.06 Longer term (13+ months): SMD −0.03 CI −0.16 to 0.10
Taylor *et al* (2005);^44^ UK	RCTs n=9 *n=1428* 24 databases, 1980–2005	COPD Patients with COPD in the community SR and meta-analysis	Conventional postdischarge care 4.5 *3–12 months*	Brief (1 month) or longer term (12 months) inpatient, outpatient or community-based interventions. All were led, coordinated or delivered by respiratory nurses via home visits, with or without telephone follow-up. Primary, secondary, community	*Readmissions*: Equivocal evidence for reduction in all-cause readmission at 12 months. One study found a 40% reduction in readmission for acute exacerbation and 57% reduction in all-cause readmission. Another found a significant reduction in readmissions. Three further studies found no effect.
Thomas *et al* (2013);^45^ UK	RCTs n=10 *Not specified* 18 databases, inception-2010	Heart failure Adult patients SR and meta-analysis	Not specified 3 *3–18 months*	Specialist HF management education: Intensive: 4–6-week appointments Decreasing intensity: every 1–2 weeks for 3 months, then every 3 months Regular: 3–4-month appointments Tailored: appointments by patient need Primary, secondary	*Admissions*: *At 3 months* (RR 0.10, 95% CI 0.01 to 0.78). *At 12 months* (5 studies), 49% reduction in relative risk (RR 0.51, 95% CI 0.41 to 0.63). *At 18 months* (1 study), no difference between groups. Interventions with *decreasing intensity* showed 58% reduction (RR 0.42, 95% CI 0.27 to 0.65). No significance for other groups.
*Chronic care model*					
Adams *et al* (2007);^46^ USA	RCTs n=32 *Not specified* 3 databases, inception-2005	COPD Adult patients with COPD SR and meta-analysis	Not specified 4 *6 weeks to 24 months*	At least one component of Wagner's CCM. Categorised according to the number of components an intervention included. Primary, secondary, community	*Admissions*: No difference in rates for interventions with 1 CCM component (n=7). Significant reduction for interventions with multiple CCM components (n=4); RR 0.78, 95% CI 0.66 to 0.94). *LoS*: *1 CCM component* (4 studies): No difference between groups. *Multiple components* (2 studies): Significant reduction in intervention (−2.51 days, 95% CI −3.40 to −1.61). *A&E use*: 3 studies with 2+ CCM components found statistically significant reduction (RR 0.58, 95% CI 0.42 to 0.79). *Costs* (7 studies): 3 RCTs showed 34% to 70% cost reduction with intervention. One RCT showed non-significant cost reductions. Three before/after studies reported an 11% to 23% reduction in costs after intervention.
de Bruin *et al* (2012);^28^ Netherlands	RCTs, CCTs, before/after, case–control n=41 *n=78 590* 6 databases, 1995–2011	Chronic disease Adult patients with multiple chronic conditions Narrative	Not specified 4.5 *Not specified*	Studies categorised by number of CCM components they included. Multiple settings, from home care organisations and community centres to primary care, hospitals, specialist clinics. Some included newly established partnerships; others provided regular care in settings where it was not normally given. Primary, secondary, community	*Admissions*: 3/16 studies found significantly reduced admissions. *Costs* (5 studies): All reported negative incremental direct healthcare costs for patients receiving intervention. Costs ranged from −US$5708 to −US$204 per patient per year, primarily due to lower inpatient costs in the intervention group.
Gonseth *et al* (2004);^47^ Spain	RCTs, CCTs, n=27 *Not specified* 3 databases, inception-2003	Heart failure Over 65s with principle or secondary diagnosis of HF SR and meta-analysis	Care without a CCM component 4.5 *3–48 months*	Education, counselling, diet advice, self-care support, discharge planning, focus on hospital to home transition, medication management, clinic review, GP follow-up. Most delivered by nurses. Varied timing (eg, in-hospital or postdischarge), organisation (eg, home care or outpatient clinic visit), duration (from single home visit to intensive intervention lasting 12 months). Primary, secondary, community	*Readmissions*: Reduced regardless of follow-up length or whether intervention delivered at home or in clinic setting. *All-cause* (6 studies): 15% reduction in readmissions (RR 0.85, 95% CI 0.79 to 0.92). *HF-specific* (6 studies): 30% reduction in readmissions (RR 0.70, 95% CI 0.62 to 0.79). *Costs* (11 studies): 10 estimated the intervention reduced costs. One reported similar costs in intervention and usual care groups.
Hisashige (2013);^69^ Japan	SR and meta-analyses n=28 *Not specified* 9 databases, 1995–2010	Chronic disease Adult patients Review of reviews	Not specified 3.5 *Not specified*	All interventions had 1+ CCM component. Typically multidisciplinary approaches with clinical follow-up by specialists, home visits, hospital discharge planning or postdischarge follow-up, counselling in hospital and patient education or reminders. Primary, secondary, community	*Admissions* (22 studies): ‘Improvement with a reasonable amount of evidence’ with intervention seen in 63% of studies (14/22). *Costs* (16 studies): 6/16 (38%) observed ‘improvement in costs with a reasonable amount of evidence’. Costs tended to focus on healthcare costs and typically did not include estimates of intervention costs.
Kruis *et al* (2013);^48^ Netherlands	RCTs n=26 *n=2997* 5 databases, 1990-present	COPD Adult patients with clinical diagnosis of COPD SR and meta-analysis	Regular follow-up visits to healthcare providers 5 *3–24 months*	Multidisciplinary (2+ providers), multitreatment (2+ CCM components), 3+ months duration. Categorised as: Exercise dominant Self-management dominant Structured nurse/GP follow-up Exercise and self-management Self-management+structured follow-up Individually tailored education Primary, secondary, community	*Admissions*: *All-*cause: number of participants with one or more admissions over 3–12 months was 27 per 100 in control vs 20 per 100 in intervention (OR 0.68, 95% CI 0.47 to 0.99, p=0.04). *Respiratory related*: at 3 months (7 studies), significant reduction (0.68, 95% CI 0.47 to 0.99, p=0.04). At 12 months (1 study), no difference observed. *LoS*: Significantly lower in the intervention group. Mean difference −3.78 days (95% CI −5.90 to −1.67, p<0.001).
Lemmens *et al* (2009);^49^ Netherlands	RCTs, before/after n=36 *Not specified* 2 databases, 1995–2008	COPD Adult patients with asthma or COPD SR and meta-analysis	Care with 0 or 1 CCM components 5 *6 weeks to 24 months*	Patient education+case management Patient education+case management+professional education Patient education with substitution of physician by nurse Professional and patient education combined with pharmacists having an active role in patient monitoring Primary, secondary, community	*Readmissions*: *Group 1* (6 studies): 1 showed significant reduction *Group 2* (6 studies): 3 showed significant reduction *Group 3*: No differences between groups *Group 4* (8 studies): Ambiguous results in all studies.
Peytremann-Bridevaux *et al* (2008);^50^ Switzerland	RCTs, controlled before/after n=13 *n=8179* 5 databases, inception-2006	COPD Adult patients undergoing disease management SR and meta-analysis	Care without a CCM component 4 *12 months*	All included 2+ CCM components; at least 1 component must have lasted 12 months. Delivered by 2+ health professionals, eg, respiratory nurse, physiotherapist, GP, practice nurse, social worker, case manager, pulmonary care physician. Primary, secondary, community	*Admissions* (10 studies): 7 showed significant effects in favour of intervention; 3 found no reduction in admissions.
Steuten *et al* (2009);^29^ Netherlands	Any with data at two time points n=20 *Not specified* 2 databases, 2005–2007	COPD Mild, moderate, severe or very severe COPD Narrative	Care without a CCM component 3.5 *2–24 months*	All included 2+ CCM components. All included self-management and delivery system redesign. Several programmes additionally encompassed decision support and/or clinical information systems Primary, secondary, community	*Readmissions*: 8/15 studies reported a reduction in readmission rates (3 statistically significant). Relative risk of readmission ranged from 0.64 to 1.50. Statistically significant improvements all seen in studies with 3 or 4 intervention components. Studies with fewer components showed no significant reductions. *Costs* (3 studies): Differences found in individual domains, eg, higher prescription costs, lower hospital costs, reduced sick leave costs. No studies reported statistically significant findings.
Woltmann *et al* (2012);^51^ USA	RCTs n=78 *Not specified* 6 databases, inception-2011	Mental health Adult patients with mental health problems SR and meta-analysis	Not specified 5 *3–36 months*	Eligible interventions had to have at least 3 CCM components. Primary, secondary, community	*Costs* (21 studies): 10 reported p values. 9 of these reported no difference between intervention and control groups; 1 favoured control condition. No statistically significant findings in any study.
*Discharge management*					
Bettger *et al* (2012);^30^ USA	RCTs, observational, n=44 *Not specified* 4 databases, 2000–2012	Stroke, Cardiac Patients hospitalised for stroke/MI Narrative	Not specified 4 *Not specified*	Hospital-initiated discharge support Community-based support models Provided by nurses, social workers, OTs, physicians, MDT. Delivered in person, in home/clinic or by telephone. Secondary, community	*Readmissions*: *Hospital-initiated support:* No impact on readmission rates in 6 studies focusing on stroke; no impact in 3 studies focusing on MI *Community-based support:* 1/4 stroke studies found significant reduction in readmissions; 5/5 MI studies found statistically non-significant trends towards reduced readmission rates
Brady *et al* (2005);^31^ Canada	Cost analyses, economic evaluations n=15 *n=6201* 6 databases, 1995–2002	Stroke Adult patients with clinical definition of stroke Narrative	Standard hospital discharge and rehabilitation 4 *Up to 12 months*	Stroke unit care and rehabilitation with specialised teams of physicians ESD with organised interdisciplinary teams to support patients at home Community rehabilitation via hospital outpatient clinics or home-based therapy Secondary, community	*Costs*: *Stroke unit care* (3 studies): Costs 3% to 11% lower (significant). *ESD* (6 studies): Non-significant trends towards costs of 4% to 30% lower for patients with mild/moderate disability. Two lower quality studies found ESD to cost more than usual care. *Community rehabilitation* (4 studies): 2 reported non-significant higher costs in intervention; 1 showed no difference, 1 reported mean direct cost to be 38% lower than day hospital rehabilitation.
Fearon *et al* (2012);^52^ UK	RCTs n=14 *n=1957* Multiple databases to 2012	Stroke Adult patients admitted to hospital with stroke SR and meta-analysis	Standard discharge arrangements 5 *3–12 months*	MDT meeting regularly, coordinated discharge, postdischarge care and rehabilitation and care at home As above, but care handed over to existing community agencies for support after immediate postdischarge period Patients access to MDT in hospital until discharge, then care provided by community stroke services Medical, nursing, physiotherapy, OT, speech and language therapists. Secondary, community	*Readmissions* (7 studies): readmission rates similar in intervention to usual care (31% vs 28%). *LoS* (13 studies): Pooled results showed significant reduction (p<0.0001). Reduction more marked in hospital outreach group than community inreach group but not statistically significant (p=0.24). *Costs* (7 studies): Overall, costs ranged from 23% less for ESD group to 15% more compared to control. No subgroup cost analyses possible.
Feltner *et al* (2014);^53^ USA	RCTs n=47 *Not specified* 5 databases, 2007–2013	Heart failure Adult patients with moderate to severe HF SR and meta-analysis	Standard discharge arrangements 4 *3–6 months*	At least one of: Patient/caregiver education Multidisciplinary HF clinic visits Home visits by nurse or pharmacist Telemonitoring Structured telephone support Transition coach/case management Interventions for provider continuity Secondary, community	*Readmissions*: *Home visits* (2 studies): Significant reduction in 30-day all-cause readmissions (RR 0.34, 95% CI 0.19 to 0.62) and 3–6-month all-cause readmissions (RR 0.75, 95% CI 0.68 to 0.86). Significant reduction in 3–6-month HF-specific readmissions (1 study), (RR 0.51, 95% CI 0.31 to 0.82). *Multidisciplinary HF clinics* (2 studies): Significant reduction in 3 to 6-month all-cause readmission (RR 0.70, 95% CI 0.55 to 0.89). No other intervention group had any significant benefits.
Jeppesen *et al* (2012);^54^ Norway, UK, Australia	RCTs n=8 *n=870* 7 databases, inception-2010 1 inception-2012	COPD Adult COPD patients in ED with acute exacerbation SR and meta-analysis	Standard discharge arrangements 4.5 *6 months*	Hospital at home: regular home visits by a trained respiratory nurse supported by the hospital team and telephone support. Secondary, community	*Readmissions* (8 studies): Significant reduction in intervention group. 9 fewer readmissions per 100 compared to inpatient care (RR 0.76, 95% CI 0.59 to 0.99, p=0.04). *Costs* (3 studies): 2 reported significant reduction in direct costs for intervention; 1 reported non-significant reduction. Authors stress low quality of economic evidence.
Lambrinou *et al* (2012);^55^ Greece	RCTs n=19 *Not specified* 3 databases, 2001–2009	Heart failure Adult patients with HF SR and meta-analysis	Standard discharge arrangements 4 *3–35 months*	Nurse-driven predischarge phase, incorporating discharge planning or inpatient education and/or evaluation. Telephone follow-up; HF clinic follow-up; home follow-up or a combination. Secondary, community	*Readmissions*: *All-cause*: Significantly reduced across all interventions (RR 0.85, 95% CI 0.76 to 0.94). Telephone, HF clinic, combined settings all non-significant. Home follow-up: RR 0.80 (95% CI 0.70 to 0.91). *HF-specific*: Significantly reduced across all interventions (RR 0.68, 95% CI 0.53 to 0.86). Telephone follow-up (RR 0.65, 95% CI 0.43 to 1.00) HF clinic: Non-significant. Home follow-up: RR 0.51 (95% CI 0.33 to 0.79) Combined settings: RR 0.58 (95% CI 0.45 to 0.73).
Langhorne *et al* (2005);^56^ UK	RCTs n=11 *n=1597* Databases not specified	Stroke Inpatients with clinical diagnosis of stroke SR and meta-analysis	Standard hospital discharge and rehabilitation 5 *3–12 months*	ESD team coordination and delivery; MDT coordinate discharge and postdischarge care and rehabilitation at home ESD team coordination; postdischarge care by community agencies No ESD team; MDT care in hospital, postdischarge care by uncoordinated community services/healthcare volunteers Medical staff, nurses, physiotherapy, therapists, assistant staff, social workers Secondary, community	*Readmissions* (5 studies): similar rates between intervention and control (27% vs 25%; OR 1.14, 95% CI 0.80 to 1.63). *LoS* (9 studies): Overall, significant reduction in intervention of 7.7 days (95% CI 4.2 to 10.7). Reduction greater for hospital outreach than community inreach (15 days, 95% CI 9 to 22 vs 5 days, 95% CI 1 to 9). Controlling for stroke severity, greater reduction in severe vs moderate group (28 days, 95% CI 15 to 41 vs 4, 95% CI 2 to 6). *Costs* (5 studies): Intervention costs lower than control (range 4% to 30% lower; median reduction 20%). Significance not stated.
McMartin (2013);^57^ Canada	RCTs, SR, meta-analysis n=11 *Not specified* 6 databases, 2004–2011	Chronic disease Adults with chronic diseases SR and meta-analysis	Standard discharge arrangements 3 *Not specified*	Discharge planning vs usual care Comprehensive discharge planning with postdischarge support vs usual care, where postdischarge support could include home visits, telephone follow-up. Secondary, community	*Readmissions*: *Discharge planning* (11 studies): Moderate evidence that intervention is effective (RR 0.85, 95% CI 0.74 to 0.97). *Discharge planning+postdischarge support*: low quality evidence that this is more effective than discharge planning alone. *LoS*: Discharge planning more effective than usual care (mean reduction of 0.91 days, 95% CI 1.55 to 0.27). Discharge planning plus postdischarge support not more effective than discharge planning alone (mean reduction 0.37 days (95% CI 0.15 to 0.60).
Olson *et al* (2011);^32^ USA	RCTs, observational, registries n=62 *Not specified* 4 databases, 2001–2011	Stroke, cardiac Adults discharged after acute stroke or MI Narrative	No transitional care across multiple providers 3.5 *12 months*	Hospital-initiated discharge support Community-based support models Chronic disease management models Patient education, goal-setting Nurses, social workers, OTs, physicians, MDT to facilitate transition from hospital to home. In person, home/clinic or telephone. Secondary, community	*Readmissions*: *Hospital-initiated support*: (8 studies): 4 studies reported reduced readmission rates; 4 reported no difference between groups. No other intervention type showed any significant difference between groups.
Phillips *et al* (2004);^58^ USA	RCTs n=19 *Not specified* 7 databases, inception-2003	Heart failure Older patients with congestive heart failure SR and meta-analysis	Standard discharge arrangements 5 *3–12 months*	Postdischarge support as: Single home visit for HF education Increased clinic follow-up Frequent telephone contact for education, self-care, appointments Extended multidisciplinary home care Day hospital service in specialist HF unit Secondary, community	*Readmissions*: *Group 1* (3 studies): 41% intervention, 53% control. Significant. (RR 0.76, 95% CI 0.63 to 0.93). *Group 2* (4 studies): 41% intervention, 41% control. Non-significant. (RR 0.64, 95% CI 0.32 to 1.28). *Group 3* (6 studies): 38% intervention, 49% control. Significant. (RR 0.79, 95% CI 0.69 to 0.91). *Group 4* (4 studies): 30% intervention, 36% control. Non-significant. *Group 5* (1 study): 7% intervention, 33% control. Significant. (RR 0.25, 95% CI 0.21 to 0.44). *LoS* (10 studies): Pooled analysis showed no significant difference between groups (mean days 8.4 vs 8.5, p=0.60). *Costs* (8 studies): 4 US based studies found significant costs reductions per patient per month of US$536 (95% CI −US$956 to −US$115). 4 non-US studies found no significant cost differences.
Phillips *et al* (2005);^59^ USA	RCTs n=7 *n=949* 5 databases, inception-2004	Heart failure Adult patients with heart failure SR and meta-analysis	Not specified 4 *3–12 months*	Specialist nurse-led clinics to manage discharge transitions. Categorised by: Complex interventions: discharge planning, postdischarge follow-up, no delay in continuity after discharge (3 studies) Less complex: no discharge planning and/or fewer components (4 studies) Secondary, community	*Readmissions*: *All-cause*: ‘Complex’ programmes non-significant (RR 0.30, 95% CI 0.04 to 2.60). ‘Less complex’ non-significant (RR 1.00, 95% CI 0.86 to 1.17). *HF-specific*: ‘Complex’ programmes significant reduction (RR 0.09, 95% CI 0.10 to 0.65. ‘Less complex’ significant reduction (RR 0.65, 955 CI 0.43 to 1.00). *LoS*: Complex interventions reduced LoS by 0.26 days compared to usual care (non-significant). Less complex interventions reduced LoS by 0.09 days (non-significant). *Costs*: Only reported for complex interventions. 3 studies showed non-significant potential savings of US$277 per patient per month.
Prieto-Centurion (2014);^33^ USA	RCTs n=5 *n=1393* 4 databases, inception-2013	COPD Exacerbation in previous 12 months Narrative	Not specified 3 *6 or 12 months*	Predischarge, postdischarge or bridging interventions across both periods. Education, health counselling, action plans delivered via telephone, home visits or consultation with primary care providers Primary, secondary, community	*Readmissions*: *All-cause*: 2/5 studies showed significant reduction at 12 months: 45% vs 67% hospitalised (p=0.028). *COPD-specific*: 1/5 studies showed significant reduction at 12 months: 32% vs 50% hospitalised (p=0.01).
Tummers *et al* (2012);^34^ Netherlands	RCTs, CCTs, n=15 *n=3536* 2 databases, inception-2011	Stroke Adult patients who had stroke Narrative	Standard hospital discharge and rehabilitation 3 *3–12 months*	Interventions grouped according to: ESD by MDT, home-based rehabilitation Stroke unit care with MDTs to reach rehabilitation goals before discharge Stroke service via network of providers organising services in all follow-up stages Primary, secondary, community	*Costs*: *Group 1* (4 studies): 3 reported non-significant increases in intervention; 1 reported no difference between groups. *Group 2* (2 studies): Both found stroke units to be more expensive than conventional care (borderline significance). *Group 3* (3 studies): 2 reported a cost reduction in intervention group.
Winkel *et al* (2008);^35^ Denmark, Sweden	RCTs n=17 *n=1122* 5 databases, inception-2005	Stroke Adult patients who had been living at home before a stroke Narrative	Standard discharge arrangements 4 *1–12 months*	Delivered by MDTs which all included physiotherapists and OTs. Some also included nurse, social worker, GP and other specialist expertise, eg, geriatrician. ESD with hospital teams providing home rehabilitation after discharge ESD with no direct rehabilitation from hospital teams Community-based rehabilitation after discharge Primary, secondary, community	*Readmissions*: *Group 1* (3 studies): No difference between groups. *Group 2* (2 studies): No difference between groups. *Group 3* (1 study): No difference between groups. *Costs*: *Group 1* (2 studies): Intervention costs significantly lower than control at 3 and 12 months. *Group 2* (1 study): ‘Some’ evidence that intervention costs are lower than control in 12 months after stroke. *Group 3* (1 study): Costs for the most independent patients were lowest when rehabilitated in hospital rather than home. Interventions most cost-effective when delivered by hospital MDT.
Yu *et al* (2006);^36^ Hong Kong	RCTs n=21 *n=4445* 3 databases, 1995–2005	Heart Failure Adult patients with heart failure Narrative	Not specified 4 *3–50 months*	Postdischarge interventions delivered via home visits, HF clinic visits and/or telephone. Interventions comprised multidisciplinary care, case management and structured discharge planning and all included patient education and/or self-management Primary, secondary, community	*Readmissions*: 11 ‘effective’ programmes had significant reductions ranging from 29% to 85%. 10 others demonstrated no significant changes. Effective programmes included an in-hospital phase, patient education, self-care, surveillance and deterioration management. Involvement of cardiac nurses and cardiologists associated with increased likelihood of successful intervention. *Costs*: 8 ‘effective’ programmes did cost analysis, 7 of which showed a cost saving for the intervention over usual care.
*Complex interventions*					
Dickens *et al* (2014);^60^ UK	RCTs n=32 *n=3941* 5 databases, inception-2013	COPD Adult patients with COPD SR and meta-analysis	Not specified 4 *1–24 months*	Multiple components and/or multiple professionals, given individually or in groups, or using technology. Could include education, rehabilitation, psychological therapy, social or organisational interventions. Delivered at home, in community, hospital or doctor clinic or combination of these. Primary, secondary, community	*A&E use*: Pooled effects showed interventions associated with 32% reduction (OR 0.68, 95% CI 0.57 to 0.80). Subgroups: *General education* (28 studies): OR 0.66, 95% CI 0.55 to 0.81. *Exercise* (11 studies): OR 0.60, 95% CI 0.48 to 0.76. *Relaxation* (4 studies): OR 0.48, 95% CI 0.33 to 0.70. Non-significant trends for interventions including skills training (p=0.35, 13 studies), relapse prevention (p=0.12, 11 studies).
Martinez-González *et al* (2014);^70^ Switzerland	SR, meta-analyses n=27 *Not specified* 4 databases, inception-2012	Chronic disease Adult patients with chronic diseases Review of reviews	Not specified 3 *Not specified*	Included any interventions based on disease management, case management, managed care, comprehensive care, multidisciplinary care, coordinated care, team care, CCMs. Primary, secondary, community	*Admissions*: 10/17 reviews demonstrated reduced admissions *Readmissions*: 7/12 reviews demonstrated reduced readmissions *LoS*: 9/13 reviews demonstrated shorter length of stay *A&E use*: 6/11 reviews showed reduced rates of ED visits *Costs*: 3/17 reviews demonstrated cost reductions
Takeda *et al* (2012);^61^ UK	RCTs n=25 *n=5942* 10 databases, inception to 2009	Heart failure Adults with at least one HF secondary care admission SR and meta-analysis	Not specified 5 *6–24 months*	All led by professionals from secondary or tertiary care. Interventions grouped as: Case management, telephone and home visits Specialist nurse-led HF clinics Multidisciplinary interventions to bridge the gap between acute and home settings Secondary, community	*Readmissions*: *HF-specific* (12 studies): Overall, significantly reduced (OR 0.57, 95% CI 0.43 to 0.75, p<0.0001). Subgroups: *Group 1*: Significant reduction at 6 months (3 studies) and 12 months (7 studies). OR 0.64 (95% CI 0.46 to 0.88) and OR 0.47 (95% CI 0.30 to 0.76), respectively. *Group 2:* No difference between groups. *Group 3* (2 studies): Significant reduction OR 0.45, 95% CI 0.28 to 0.72). All-cause also significantly reduced with multidisciplinary interventions: (OR 0.46, 95% CI 0.30 to 0.69).
*Multidisciplinary teams*					
Health Quality Ontario (2012);^71^ Canada	SR and meta-analyses n=24 *Not specified* 6 databases, 2008–2011	Heart failure, COPD Adult patients with heart failure or COPD Review of reviews	Usual care in general practice 3 *Not specified*	Interventions to provide formalised links between primary and specialist care via disease-specific education, medication review, physical activity and lifestyle counselling, self-care and follow-up. Delivered by intermediate care teams including GPs, specialists, nurses, social workers, pharmacists, dieticians. Primary, secondary, community	*Admissions*: *All-cause* (7 studies). Non-significant 4% RR reduction after 1 year (low quality). *COPD-specific* (4 studies). Significant 25% RR reduction after 1 year (moderate quality). *HF-specific* (6 studies). Non-significant 14% RR reduction after 1 year (low quality).
Health Quality Ontario (2013);^37^ Canada	SR, RCTs, observational studies n=20 *Not specified* 5 databases, 2002–2011	Chronic disease Adult patients with one or more chronic diseases Narrative	Not specified 3 *Not specified*	Informational, management and relational continuity. Assessed by: Duration (length of relationship) Density (number of visits with same provider in a set period) Dispersion (visits with distinct providers) Sequence (order of seeing providers). Primary, community	*Admissions*: Three studies. None reported any significant differences between intervention and control groups (low quality).
Holland *et al* (2005);^62^ UK	RCTs n=30 *n=8158*13 databases inception-2004	Heart failure Adult patients with congestive heart failure SR and meta-analysis	Not specified 5 *Not specified*	Interventions with management by an MDT that included medical input plus one or more of specialist nurse, pharmacist, health educator, dietician or social worker: Education/self-management home visits Telephone follow-up only Intervention during hospital admission or hospital clinic attendance Primary, secondary, community	*Admissions*: *All-cause* (21 studies): Significant reduction in intervention (RR 0.87, 95% CI 0.79 to 0.95, p=0.002). Significant heterogeneity. *HF-specific* (16 studies): Significant reduction in intervention (RR 0.70, 95% CI 0.61 to 0.81, p<0.0001). *LoS* (10 studies): Significant reduction in mean inpatient days of 1.9 in intervention (95% CI 0.71 to 3.1). Home-based interventions reduced mean days in hospital. Interventions solely delivered in hospital, clinic or primary care showed no significant benefits.
Koshman *et al* (2008);^63^ Canada	RCTs n=12 *n=2060* 10 databases inception-2007	Heart failure Adult patients with heart failure SR and meta-analysis	Heart failure care without pharmacist involvement 4 *6–12 months*	Pharmacists providing HF and medication education through self-monitoring support, compliance facilitation. Either via directed care where pharmacist is the key driver, or collaborative care with pharmacist as part of MDT. Secondary, community	*Admissions*: *All-cause* (11 studies): Significant reduction (OR 0.71, 95% CI 0.54 to 0.94). No difference between directed and collaborative care model. *HF-specific* (11 studies): Significant reduction (OR 0.69, 95% CI 0.51 to 0.94). Collaborative care model associated with greater reduction in HF-specific admission than directed care (OR 0.42, 95% CI 0.24 to 0.74 vs OR 0.89, 95% CI 0.68 to 1.17, p=0.02).
McAlister (2004);^64^ UK	RCTs n=29 *n=5039* 7 databases, inception-2003	Heart failure Adult patients with HFSR and meta-analysis	Not specified 4 *1–12 months*	Multidisciplinary HF clinic MDT providing specialised follow-up outside hospital Telephone follow-up with primary care attendance in the event of deterioration Self-care education Primary, community	*Admissions*: *Groups 1+2:* HF hospitalisation significantly reduced (RR 0.74, 95% CI 0.63 to 0.87); all-cause hospitalisation significantly reduced (RR 0.81, 95% CI 0.71 to 0.92). *Group 3:* HF hospitalisation significantly reduced (RR 0.66, 95% CI 0.52 to 0.83). All-cause hospitalisation no significant effect. *Group 4:* HF hospitalisation significantly reduced (RR 0.66, 95% CI 0.52 to 0.83). All-cause hospitalisation significantly reduced (RR 0.73, 95% CI 0.57 to 0.93). *Costs* (18 studies): 15 found cost savings; 3 found neutral costs.
Medical Advisory Secretariat (2009);^65^ Canada	RCTs n=8 *n=2692* 4 databases, inception-2008	Heart failure Adult patients with HF SR and meta-analysis	Care not provided by multiple practitioners 4 *At least 12 months*	All included a team of nurse and physician and/or general practitioner, one of which specialised in HF management. Varying combinations of disease-specific education, diet, lifestyle, exercise counselling, self-care support, follow-up. Delivered directly (clinic based programme) or indirectly (telephone based, physician supervised, nurse-led). Primary, secondary, community	*Readmissions*: *All-cause* (7 studies): Non-significant *increase* in intervention group. Significant 12% *reduction* when care delivered through a direct (clinic) model. *HF-specific* (6 studies): Non-significant RR reduction of 14% in intervention. *LoS* (7 studies): Patients receiving intervention generally had shorter LoS whether measured as mean duration (4 studies) or total bed days (3 studies). *A&E use* (1 study): 77% of intervention patients vs 84% of control patients had an ED visit within 12 months (p=0.029).
Roccaforte *et al* (2005);^66^ Canada	RCTs n=33 *Not specified* 4 databases, 1980–2004	Heart failure HF patients followed up in outpatient setting SR and meta-analysis	Referral to family physician or home care services after discharge 5 *3–22 months*	Multidisciplinary approach, starting during hospitalisation, continuing for up to 12 months postdischarge, delivered by various professionals Approach centred on specific health professionals, eg, HF specialist nurses or case managers, focused on particular care components, eg, therapy adherence Primary, secondary, community	*Readmissions*: *All-cause*: 7/32 studies found significant reductions (OR 0.76, 95% CI 0.69 to 0.94). *HR-specific*: 8/20 found significant reductions (OR 0.58, 95% CI 0.50 to 0.67). By subgroup: *Group 1:* All-cause and HF-specific readmissions significantly reduced (OR 0.58, 95% CI 0.47 to 0.71) and (OR 0.58, 95% CI 0.45 to 0.75), respectively. *Group 2:* All-cause and HF-specific readmissions significantly reduced (OR 0.82, 95% CI 0.74 to 0.91) and ()R 0.61, 95% CI 0.51 to 0.73), respectively. *LoS* (12 studies): Significant reduction of −1.49 days (95% CI −2.03 to −0.95 days).
Sikich (2012);^38^ Canada	HTAs, SR, RCTs, n=6 *n=1370* 6 databases, 1995–2010	COPD Adult patients with COPD Narrative	Care not provided by multiple practitioners 4 *3–12 months*	Interventions based on CCM components, delivered by various professionals as a team in one organisation or range of organisations together as a unique team. Most teams included a respiratory specialist and/or a physician. Primary, secondary, community	*Admissions*: *All-cause* (4 studies): Statistically significant 25% RR reduction in favour of intervention (p<0.0001) (moderate evidence). *COPD-specific* (3 studies): Statistically significant 33% RR reduction in favour of intervention (p=0.002) (moderate evidence). *A&E use*: *All-cause* (2 studies): Both showed non-significant reduction (RR 0.64, 95% CI 0.31 to 1.33). *COPD-specific* (1 study): Significant reduction (RR 0.59, 95% CI 0.43 to 0.81).
Smith *et al* (2007);^39^ Ireland	RCTs, CCTs, before/after, time series n=20 *Not specified* 7 databases, inception-2006	Chronic disease Patients in a primary and secondary shared care service Narrative	Care not provided by multiple practitioners 5 *Not specified*	Liaison meetings attended by specialists and primary care staff to discuss and plan ongoing patient management; shared care record carried by the patient, computer-assisted shared care and email with data available to primary and secondary care Primary, secondary	*Admissions* (7 studies): Mixed results. Intervention was associated with a reduction in admissions in older patients and those with higher baseline morbidity. *Costs* (11 studies): 3 performed full economic analyses, of which 2 reported incremental cost savings in intervention. Seven studies reported direct costs: 1 showed higher costs in intervention; 6 reported mixed results (4/6 showed intervention more expensive than control, 2/6 reported lower costs in intervention).
Smith *et al* (2012);^40^ Ireland*	RCTs, CCTs, before/after, time series n=10 *n=3357* 9 databases, various–2011	Chronic disease Patients with multimorbidity in primary care or community Narrative	Not specified 5 *2–24 months*	Any intervention to improve outcomes for patients with multimorbidity in primary or community care delivered by an MDT. 6 studies assessed MDT interventions. Primary, community	*Admissions* (5 studies): One study found significant reduction in admissions with intervention; 4 found no difference between groups. *Costs* (4 studies): One reported no difference between groups; one had no results available; one reported a non-significant marginal benefit for intervention, one reported net savings in intervention costs but did not account for other costs.
*Self-management*					
Franek (2013);^41^ Canada	SR, RCTs, meta-analyses n=10 *n=6074* 5 databases, 2000–2012	Chronic disease Adult patients with chronic disease Narrative	Care from the usual provider 3.5 *4–12 months*	Stanford chronic disease programme: 6 weekly 2.5 hour sessions with 10–15 participants, in community settings, with volunteer lay facilitators assisting patients to make their own management choices and reach self-selected goals. Primary, secondary, community	*Admissions* (3 studies): No significant difference in admission rates between intervention and control in any study (low quality evidence). *LoS* (5 studies): None showed any significant differences between groups at 6 months. *A&E use* (5 studies): No significant differences between groups.
Harrison *et al* (2015);^67^ Canada	RCTs n=7 *n=1115* 7 databases, inception-2014	COPD Adult patients hospitalised following acute exacerbation SR and meta-analysis	Not specified 3.5 *2 weeks-12 months*	Action plans involving symptom monitoring, education and at least 2 of 7 self-management skills (self-efficacy, problem solving, resource use, collaboration, emotional/role management, goal setting). Delivered by nurses when patient is in hospital, or within 1 month of discharge. Secondary, community	*Readmissions* (5 studies). Meta-analysis found no significant differences at 12 months between intervention and control groups in terms of the number of patients readmitted to hospital. Mean difference 1.32, CI 0.71 to 2.46 (p=0.38).
Jovicic *et al* (2006);^42^ Canada	RCTs n=6 *n=857* 6 databases, inception-2005	Heart failure Adult patients with HF Narrative	Not specified 4 *3–12 months*	Education and limited follow-up: patients taught to monitor condition and recognise symptom exacerbation; follow-up phone call and face to face or digital education. Delivered by nurses or AHPs. Secondary, community	*Readmissions*: *All-cause* (5 studies): Significant reduction in intervention (OR 0.59, 95% CI 0.44 to 0.80). *HF-specific* (3 studies): Significant reduction in intervention (OR 0.44, 95% CI 0.27 to 0.71). *Costs* (3 studies): All reported annual savings for intervention vs usual care of between US$1300 and US$7515.
Smith *et al* (2012);^40^ Ireland	RCTs, CCTs, before/after, time series n=10 *n=3357* 9 databases, various–2011	Chronic disease Patients with multimorbidity in primary care or community Narrative	Not specified 5 *2–24 months*	Any patient-orientated intervention to promote self-management in patients with multimorbidity in primary or community care. Four studies assessed self-management interventions. Primary, community	*Admissions* (2 studies): One reported significant reduction in favour of intervention. The other found no difference between groups. *Costs* (2 studies): One reported cost savings per participant due to reduction in admission rates in intervention group. The other found no difference between groups.
Zwerink *et al* (2014);^68^ Netherlands	RCTs, controlled trials, n=31 *n=3688* 6 databases, 1995–2011	COPD Patients with clinical diagnosis of COPD SR and meta-analysis	Not specified 5 *2–24 months*	Structured interventions to improve self-health and self-management skills. At least 2 of action plan, exercise programme, smoking cessation, dietary advice, medication review, coping with breathlessness advice, CBT, motivational interviewing, goal setting, feedback. Primary, secondary, community	*Admissions*: *All-cause* (6 studies): 310 patients per 1000 admitted within 12 months in intervention vs 428 control. Statistically significant reduction (OR 0.60, 95% CI 0.40 to 0.89). *COPD-specific* (9 studies): 190 patients per 1000 admitted within 12 months in intervention vs 293 control. Statistically significant reduction (OR 0.57, 95% CI 0.43 to 0.75). *LoS* (5 studies): No differences between groups.

*Smith *et al*^40^ listed twice due to focus on MDT interventions and self-management interventions.

AHP, allied health professional; CBT, cognitive–behavioural therapy; CCM, chronic care model; CCT, controlled clinical trial; ED, emergency department; ESD, early supported discharge; HF, heart failure; HTA, health technology assessment; LoS, length of stay; MDT, multidisciplinary team; MI, myocardial infarction; OT, occupational therapy; QA, quality assessment; RR, relative risk; SMD, standardised mean difference; SR, systematic review.

The most commonly studied condition was chronic disease (n=15),^23–28^ ^37^ ^39–41^ ^43^ ^57^ ^69^ ^70^ followed by heart failure (n=14),^36^ ^42^ ^45^ ^47^ ^53^ ^55^ ^58^ ^59^ ^61–66^ COPD (n=12),^29^ ^33^ ^38^ ^44^ ^46^ ^48–50^ ^54^ ^60^ ^67^ ^68^ stroke (n=5),^31^ ^34^ ^35^ ^52^ ^56^ stroke and cardiac conditions (n=2),^30^ ^32^ mental health (n=1)^51^ and heart failure and COPD combined (n=1).^71^ All reviews were published between 2004 and 2015. Reviews were published in Canada,^26^ ^31^ ^37^ ^38^ ^41^ ^42^ ^57^ ^63^ ^65–67^ ^71^ the UK,^24^,^43–45^ ^52^ ^56^ ^60–62^ ^64^ the USA,^23^ ^30^ ^32^ ^33^ ^46^ ^51^ ^53^ ^58^ ^59^ the Netherlands,^25^ ^27–29^ ^34^ ^48^ ^49^ ^68^ Ireland,^39^ ^40^ Switzerland,^50^ ^70^ Norway,^54^ Japan,^69^ Hong Kong,^36^ Spain,^47^ Denmark^35^ and Greece.^55^ In most reviews, the comparator was usual clinical care, although a detailed description of usual care was typically not provided. Overall, 29 reviews (58%) reported a nominally statistically significant result for at least one outcome.

### Quality of included reviews

The mean quality assessment (QA) score was 4/5. Twelve reviews scored 5/5 (24%).^39^ ^40^ ^48^ ^49^ ^51^ ^56^,^58^ ^61^ ^62^ ^66^ ^68^ The criterion for which the largest number of reviews failed to score a point related to whether a valid consideration of bias across primary studies had been undertaken. There was no discernible trend in review quality across intervention categories: the mean QA scores by the intervention group ranged from 3.4/5 (case management) to 4.2/5 (chronic care model (CCM), multidisciplinary teams (MDT), self-management).

### Effects by intervention type

Interventions were categorised into six broad groups (table 3), although intervention components frequently overlapped.

**Table 3:** Intervention groupings

Category	Description of intervention
Case management (n=8)^23–27^ ^43–45^	Based on implementation of a collaborative process between one or more care coordinators or case managers and the patient, to assess, plan and facilitate service delivery for patients with chronic diseases, particularly when transitions across healthcare settings are required
Chronic care model (n=9)^28^ ^29^ ^46–51^ ^69^	Model that identifies six modifiable elements of healthcare systems: (1) organisational support, addressing organisational culture and leadership, (2) clinical information systems to organise patient, population and provider data, (3) delivery system design to address composition and function of the care team and follow-up management, (4) decision support to increase provider access to evidence-based guidelines and specialists for collaboration, (5) self-management support to provide tailored education, skills training, psychosocial support and goal-setting and (6) community resources to provide peer support, care coordination and community-based interventions
Discharge management (n=15)^30–36^ ^52–59^	Interventions designed to facilitate effective transitions from hospital care to other settings. Typically includes a predischarge phase of support, transitional care for the move between the hospital and community/home setting and postdischarge follow-up and monitoring, often incorporating rehabilitation or reablement support
Complex interventions (n=3)^60^ ^61^ ^70^	Two reviews assessed a range of interventions rather than focusing on a single intervention or service model
Multidisciplinary teams (n=10)^37–40^ ^62–66^ ^71^	Interventions comprising teams composed of multiple health and/or social care professionals working together to provide care for people with complex needs. Teams typically included condition-specific expertise, nurses, occupational therapists, physiotherapists, social workers, GPs and occasionally pharmacists or case managers
Self-management (n=5)^40–42^ ^67^ ^68^	Interventions designed to provide patient support, typically via tailored education to inform the patient about their condition(s), recognising signs and symptoms of disease exacerbation, dietary and lifestyle advice and/or condition-specific education supporting medication adherence

Eight reviews focused on case management interventions.^23–27^ ^43–45^ With the exception of one review which showed that case management was associated with significantly reduced healthcare costs,^26^ and another that demonstrated a 49% relative risk reduction (RRR) in admissions for patients with heart failure,^45^ all case management reviews showed mixed findings or no association between the intervention and outcomes assessed. Of nine reviews focusing on interventions comprising one or more components of the CCM, six reported positive findings for at least one outcome.^28^ ^46–48^ ^50^ ^69^ All CCM reviews reported that interventions with multiple components were significantly more effective than single component interventions at reducing admission rates,^46^ ^49^ ^50^ ^69^ with reductions of 22–32% observed in reviews that performed meta-analysis. Multicomponent interventions were also successful in reducing readmissions by 15–30%,^47^ length of hospital stay by 2–4 days^46^ ^48^ and A&E visits by 42%.^46^

Fifteen reviews assessed discharge management interventions, predominantly focusing on readmission rates and length of stay (LoS). Six reviews reported significant reductions in readmission rates for patients with heart failure,^53^ ^55^ ^58^ ^59^ COPD^54^ and general chronic diseases.^57^ Reductions ranged from 15%^55^ to 66%.^53^ In contrast, discharge management for patients who had stroke was notably ineffective in reducing readmission rates,^32^ ^35^ ^52^ ^56^ although LoS reduced by 7.7 days in one stroke review.^56^ Three reviews assessed complex interventions. One demonstrated a 32% reduction in A&E use,^60^ another reported a 43% reduction in heart failure-related readmissions^61^ and a review of reviews reported positive findings for admissions, readmissions, LoS and A&E use (no effect sizes given).^70^

Ten reviews assessed MDT interventions. Although team composition varied, MDT were generally effective when used for patients with single conditions, showing a 26–31% reduction in admission rates for heart failure^62–64^ and a 33% RRR for admissions in patients with COPD.^38^ MDT were also associated with a 42% reduction in heart failure readmissions,^66^ a 2-day reduction in LoS,^62^ ^65^ ^66^ significantly reduced A&E use^65^ and significantly lower healthcare costs.^64^ Conversely, MDT for general chronic disease management showed mixed effectiveness or no significant association for any outcomes,^37^ ^39^ ^40^ suggesting that the crucial component of an effective MDT is the inclusion of condition-specific specialist expertise in the team skill mix. Finally, five reviews assessed self-management interventions. Three showed either mixed findings^40^ or no association between intervention and outcomes assessed.^41^ ^67^ The remaining two demonstrated significant reductions in readmission rates and healthcare costs for patients with heart failure^42^ and significantly lower admission rates for COPD.^68^

### Hospital admissions

Emergency admission rates were assessed in 21 reviews across five intervention categories (table 4). Eleven reviews reported significantly reduced admissions,^38^ ^45^ ^46^ ^48^ ^50^ ^62–64^ ^68–70^ with all but two positive reviews focusing on heart failure^45^ ^62–64^ or COPD.^38^ ^46^ ^48^ ^50^ ^68^ The most effective interventions were based on the CCM, for which 4/5 reviews showed statistically significant reductions in admission rates following the intervention. Multiple component strategies were associated with reductions of between 22%^46^ and 32%^48^ in admission rates for patients with COPD.

**Table 4:** Summary of effectiveness for each outcome by review and intervention category

Review	Admissions	Readmissions	Length of stay	A&E use	Costs
Case management					
Hickam *et al* (2013)^23^	?				?
Hutt *et al* (2004)^24^	?		?	?	?
Latour *et al* (2007)^25^		?	?	=	
Manderson *et al* (2012)^26^					–
Oeseburg *et al* (2009)^27^	?		?	?	?
Stokes *et al* (2015)^43^					=
Taylor *et al* (2005)^44^		?			
Thomas *et al* (2013)^45^	–				
Chronic care model					
Adams *et al* (2007)^46^	–		–	–	–
de Bruin *et al* (2012)^28^	?				–
Gonseth *et al* (2004)^47^		–			–
Hisashige (2013)^69^	–				?
Kruis *et al* (2013)^48^	–		–		
Lemmens *et al* (2009)^49^		?			
Peytremann-Bridevaux *et al* (2008)^50^	–				
Steuten *et al* (2009)^29^		?			=
Woltmann *et al* (2012)^51^					=
Discharge management					
Bettger *et al* (2012)^30^		?			
Brady *et al* (2005)^31^					?
Fearon *et al* (2012)^52^		=	–		?
Feltner *et al* (2014)^53^		–			
Jeppesen *et al* (2012)^54^		–			–
Lambrinou *et al* (2012)^55^		–			
Langhorne *et al* (2005)^56^		=	–		?
McMartin (2013)^57^		–	–		
Olson *et al* (2011)^32^		=			
Phillips *et al* (2004)^58^		–	=		–
Phillips *et al* (2005)^59^		–	=		=
Prieto-Centurion *et al* (2014)^33^		?			
Tummers *et al* (2012)^34^					?
Winkel *et al* (2008)^35^		=			–
Yu *et al* (2006)^36^		?			–
Complex interventions					
Dickens *et al* (2014)^60^				–	
Martinez-Gonzelez *et al* (2014)^70^	–	–	–	–	?
Takeda *et al* (2012)^61^		–			
Multidisciplinary teams					
Health Quality Ontario (2012)^71^	–				
Health Quality Ontario (2013)^37^	–				
Holland *et al* (2005)^62^	–		–		–
Koshman *et al* (2008)^63^		?		–	
McAlister *et al* (2004)^64^	?				
Medical Advisory Secretariat (2009)^65^	=		–		
Roccaforte *et al* (2005)^66^		–	–		
Sikich (2012)^38^	–			?	
Smith *et al* (2007)^39^	?				?
*Smith *et al* (2012)^40^	?				?
Self-management					
Franek (2013)^41^	=		=	=	
Harrison *et al* (2015)^67^		=			
Jovicic *et al* (2006)^42^		–			–
Smith *et al* (2012)^40^	?				?
Zwerink *et al* (2014)^68^	–		=		

*Smith *et al* (2012) listed twice due to focus on MDT interventions and self-management interventions.

MDT interventions were also effective, with 4/8 reviews showing significant reductions in admissions. Effect sizes ranged from 25% for a COPD MDT with formal links to primary care,^38^ through 26% for teams that included specialist heart failure expertise,^64^ to 31% for teams that included pharmacists as collaborators.^63^ One review of structured self-management interventions demonstrated a 43% reduction in the relative risk of COPD-related admission.^68^ Case management interventions were largely ineffective in reducing admission rates, with 3/4 showing mixed findings,^23^ ^24^ ^27^ although one case management intervention for heart failure comprising intensive follow-up that gradually reduced in intensity over time showed a potential 58% reduction in admissions.^45^

Most reviews reported condition-specific admissions and admissions for any cause. In all cases, potential reductions in condition-specific admissions were substantially greater than those for all-cause admissions.^38^ ^45^ ^62–64^ ^68^

### Hospital readmissions

Twenty-four reviews assessed readmissions. Eleven reported positive findings: eight for heart failure,^42^ ^47^ ^53^ ^55^ ^58^ ^59^ ^61^ ^66^ two for chronic disease^57^ ^70^ and one for COPD.^54^ Discharge management was the most effective intervention, with 6/13 reviews showing significant reductions in readmission rates.^53–55^ ^57–59^ Interventions incorporating an inpatient phase and postdischarge support at home were associated with reductions in condition-specific readmission rates of 24%^32^ and 49%^53^ for heart failure interventions, 24% for a hospital at home intervention for COPD^54^ and a 15% reduction for patients with chronic diseases.^57^ Similarly, ‘complex’ interventions that included specialist nurse-led clinics for heart failure follow-up were associated with a 91% reduction in condition-specific readmission rates in one review,^59^ and postdischarge hospital outreach coordinated by a MDT was associated with a 32% reduction in heart failure readmission rates.^55^ In contrast, discharge interventions for patients who had stroke were ineffective, with 0/4 reviews assessing this intervention showing no differences between intervention and control groups.^32^ ^35^ ^52^ ^56^

Other interventions showed less comprehensive evidence. One of three CCM reviews that assessed readmissions found a 30% reduction in readmission rates for heart failure.^47^ One self-management review in which nurses provided heart failure-specific education reported a 56% reduction in readmissions.^42^ Two reviews assessing complex interventions reported significant reductions in readmission rates: one for a heart failure case management intervention^61^ and another for patients with general chronic diseases.^70^ One MDT review showed a 42% reduction in heart-failure specific readmission, with subgroup analysis indicating that heart failure specialist nurses could reduce condition-specific readmissions by up to 39%.^66^

As with admissions, potential reductions in readmissions were substantially greater for condition-specific readmissions than all-cause readmissions, with effect sizes in the former typically double those for the latter.^47^ ^53^ ^56^ ^66^

### Length of stay

Sixteen reviews assessed LoS, across six intervention categories. Neither case management interventions^24^ ^25^ ^27^ or self-management interventions^41^ ^68^ showed evidence of effectiveness, but there were positive findings in the CCM,^46^ ^48^ discharge management,^52^ ^56^ ^57^ complex intervention^60^ and MDT groups.^62^ ^65^ ^66^ Two CCM interventions were associated with a significantly reduced mean LoS for COPD of 2.51^46^ and 3.78 days, respectively.^48^

Three discharge management reviews showed significant LoS reductions. Two were for patients who had stroke, including postdischarge support coordinated through multidisciplinary hospital outreach^52^ and early supported discharge.^56^ Pooled results from the early supported discharge meta-analysis suggested a mean LoS reduction of 7.7 days, rising to 28 days for the most severely impaired patients compared to 4 days for moderately impaired patients.^56^ One discharge management intervention for patients with chronic diseases reported positive results, with a modest reduction of 0.91 days.^57^

Finally, three MDT interventions showed significant reductions in LoS, all for heart failure patients. Again, reductions were modest at 1.9 days for an MDT that included a clinician plus specialist nurse, pharmacy, health education, dietician and social worker support,^62^ a ‘generally shorter’ LoS for an intervention based on nurses, heart failure physicians and general practitioners (GPs) providing condition-specific patient education^65^ and a MDT providing hospital outreach for at least 12 months after hospital discharge was associated with a mean reduction in LoS of 1.49 days.^66^

### Accident and emergency use

Nine reviews measured the effectiveness of interventions in reducing Accident and Emergency (A&E) use. Five reviews included patients with chronic diseases, all showing mixed findings or no association between intervention and outcome.^24^ ^25^ ^27^ ^41^ ^70^ The remaining reviews assessed single conditions, with 2/3 demonstrating statistically significant reductions in A&E use for COPD,^46^ ^60^ and one showing significant findings in patients with heart failure.^65^

Case management and self-management interventions were ineffective in reducing A&E use.^24^ ^25^ ^27^ ^41^ Effective interventions related to the CCM, where multicomponent COPD interventions were associated with a 42% reduction in A&E use,^46^ the complex intervention group, where interventions with multiple components administered by multiple professionals demonstrated a potential 32% reduction in A&E use,^60^ and the MDT group, where one review found a significant reduction in A&E use when an MDT for heart failure contained condition-specific specialist expertise.^65^ However, A&E use remained high overall, with 77% of patients in the intervention group having at least one emergency department visit during the 12-month follow-up period, compared to 84% of control patients.

### Costs

Twenty-five reviews assessed healthcare costs but the evidence base was poor and heterogeneous—information on potential cost savings was typically qualitative and could not be compared across reviews. Ten reviews reported positive findings: five for patients with heart failure,^36^ ^42^ ^47^ ^58^ ^64^ two for COPD,^46^ ^54^ two for chronic disease^26^ ^28^ and one for stroke.^35^ Eleven reviews reported mixed findings, all for chronic disease^23^ ^24^ ^27^ ^39^ ^40^ ^69^ ^70^ or stroke,^31^ ^34^ ^52^ ^56^ and four reported no difference in costs between intervention and control groups for chronic disease,^43^ COPD,^28^ mental health^51^ or heart failure.^59^

The most effective interventions were based on the CCM, with three reviews reporting significantly reduced costs.^28^ ^46^ ^47^ One review reported cost savings of between 34% and 70% for CCM interventions but gave no further detail of the nature of these savings.^46^ Discharge management interventions were cost-effective in some cases,^35^ ^36^ ^54^ ^58^ predominantly due to reduced hospitalisation costs and fewer patient bed days. MDT interventions that included specialist expertise also showed some evidence for cost-effectiveness but again, little detail was given to substantiate this.^64^

## Discussion

The primary aim of this review was to assess whether integrated care—through interventions to coordinate care across two or more health and/or social care settings for patients with chronic diseases—can reduce hospital activity and if so, to what extent. Despite the diverse evidence base and variations within and across reviews in terms of the characteristics, duration and intensity of interventions, some positive trends were evident. Overall, the most effective interventions included discharge planning and postdischarge support for hospital inpatients,^53–55^ ^57–59^ MDT care—particularly when condition-specific specialists, specialist nurses or pharmacists were part of the team skill mix,^38^ ^63–65^ and interventions based on multiple components of the CCM,^28^ ^46–48^ ^50^ ^69^ although no CCM reviews reported which specific components were most likely to produce positive outcomes. Self-management showed most promise when incorporated into MDT care or when tailored patient education was included in discharge planning.^42^ ^68^ The least effective intervention was case management. Although in theory this intervention may increase health service efficiency by reducing unnecessary contacts with healthcare professionals,^43^ we found little evidence of effectiveness. Some of the key features of effective interventions are outlined in table 5. This table is not intended as a ‘toolkit’ for effectiveness, since interventions or components that reduced hospital activity for some outcomes and/or conditions were not necessarily effective for others. Nevertheless, it summarises some of the ‘ingredients’ of potentially effective integrated care interventions.

**Table 5:** Summary of intervention effectiveness

Intervention/feature*	Notes/caveats
Complex interventions with multiple components	Greatest effects when treating patients with single rather than multiple conditions No reviews stated specific components that were more (or less) likely to be effective than others
Postdischarge hospital outreach coordinated by a multidisciplinary team	Greatest effects when treating patients with single rather than multiple conditions In contrast, community inreach interventions not effective, even when featuring MDT Not effective for patients who had stroke
MDT with: Disease-specific specialists as core members Specialist nurse-led clinics Pharmacists as collaborative partners	Greatest effects when treating patients for single conditions No reviews compared the ‘added value’ that a given professional or clinician may bring to a MDT, so optimal composition remains unknown
Transition from hospital to home is most effective when interventions are initiated during the inpatient phase and continue postdischarge	
Home-based community follow-up	Effective for reducing length of stay in patients who had stroke Community follow-up least likely to be effective when delivered through a case management model
Self-management education combined with multidisciplinary approaches or discharge planning	Greatest effects when treating patients for single conditions Simple self-management interventions were largely ineffective

*MDT, multidisciplinary team.

All hospital activity outcomes showed some significant reductions. Proportionally, LoS was the most likely to reduce, with 9/16 reviews reporting positive findings. However, gains were typically modest: multicomponent CCM strategies could reduce LoS by 2.5–4 days,^46^ ^48^ and MDT care with specialist expertise was associated with LoS reduction of 1.5–2 days.^62^ ^66^ For admissions, 11/21 reviews demonstrated positive findings, suggesting potential reductions of between 15% and 50%. Readmission rates were significantly reduced in 11/24 reviews, suggesting a 10–30% reduction in all-cause readmission and a 25–50% reduction in condition-specific readmission could be achieved with interventions based on discharge management,^52–55^ MDT^66^ and the CCM.^46^ ^48^ ^50^ ^69^ A&E use typically reduced by 30–40% in reviews of effective interventions.^46^ ^60^ ^65^ It has been argued that integrated care may increase hospital activity due to supply induced demand, in which integration uncovers unmet patient need.^72^ ^73^ Several reviews noted minor increases in activity following case management,^24^ ^27^ CCM,^51^ discharge management^31^ and MDT interventions.^39^ ^65^ However, these increases were typically restricted to one or two primary studies within a review and were rarely statistically significant.

A secondary objective was to assess the settings and patient populations for which promising interventions may be most effective. Interventions focused on single conditions showed greater effectiveness than those implemented for patients with general chronic diseases. Those that assessed MDT care or discharge management for patients with heart failure and COPD were typically effective in reducing admissions,^38^ ^62–64^ readmissions^53–55^ ^58^ ^59^ and LoS,^62^ ^65^ ^66^ with some positive trends evident in reducing A&E use.^65^ This may reflect the difficulty of designing effective interventions for people with a broad range of conditions, in a healthcare system where care for patients with complex needs remains largely centred on single condition guidelines. Furthermore, interventions such as MDT have been an established feature of disease management for conditions like heart failure for a number of years, and the particular success of interventions focused on this patient group is likely to reflect this. Care offered in patients’ homes, whether following discharge from hospital,^53^ ^54^ through MDT care,^38^ ^62^ ^64^ or through self-management interventions^42^ ^68^ was significantly associated with reduced hospital activity, particularly when home care was coordinated by multidisciplinary outreach as opposed to a community in-reach model. Although these interventions were associated with significantly reduced hospital activity, the most successful were coordinated by the acute sector, suggesting that effective integrated care may still rely on the deployment of substantial hospital resources and the involvement of multiple acute sector healthcare professionals.

Our final objective was to assess the cost implications of integrated care interventions. Data were poor: the care components that cost data referred to were often unclear and effect sizes were rarely stated. Where statistical significance was described, the majority of savings appeared to come from a reduction in costs incurred through hospitalisation, whether this was because interventions allowed patients to be discharged from hospital earlier or whether interventions reduced subsequent rates of hospitalisation or rehospitalisation. As a result, interventions which included some element of home care or rehabilitation tended to be cost saving compared to care in which rehabilitation was provided within the hospital environment.^28^ ^35^ ^42^ ^46^ ^47^ ^54^ However, it is likely that substantial cost savings can only be realised if hospital capacity can be physically removed from the system, for example, through ward closures. We found little evidence of this following integrated care interventions.

### Strengths and weaknesses

This is the first umbrella review of its kind and is timely given the increasing emphasis on integrated care in healthcare policy with the key aim of reducing hospital use. By undertaking an umbrella review of systematic reviews, we could assess a large volume of evidence across diverse conditions, interventions and outcomes. However, umbrella reviews have limitations. Grouping interventions in a way that allowed meaningful conclusions to be drawn about their effectiveness was challenging. Although we employed the Effective Practice and Organisation of Care Group (EPOC) taxonomy^74^ as an organisational framework, few interventions were mutually exclusive and the characteristics and form of interventions frequently overlapped. For example, most discharge management interventions were delivered by MDT, and several interventions included some element of self-management support. Even for reviews which shared broadly similar intervention characteristics, the duration of follow-up, study design, complexity, intensity and mode of delivery varied. Furthermore, because the unit of analysis is the review rather than the primary study level, the re-synthesis of information at the umbrella review level that has already been synthesised at review level risks loss of detail or misinterpretation of findings and trends. But, by restricting inclusion to reviews receiving moderate, good or high QA scores, we reduced the likelihood of evidence misinterpretation and the incidence of discordant findings. Successful approaches to integrated care have highly context-specific histories, yet by undertaking an umbrella review, we were unable to draw conclusions about the specific contexts in which interventions were implemented. Nevertheless, we believe that the methodological strengths of our approach outweigh the limitation of being unable to comment on the specific contexts in which interventions were implemented.

### Implications for clinicians and policymakers

Although there was evidence that some integrated care interventions can reduce hospital activity, effects were rarely unequivocally positive. The size of gains from integration may also be modest.^17^ For example, in recent years, the trend in outcomes such as length of hospital stay has been steadily reducing, largely due to improved surgical techniques and increased day case treatment.^8^ This suggests that there may be limits to the absolute reductions in key hospital activity metrics that integrated care initiatives could achieve.^1^ This was evident in several reviews that noted statistically significant differences in outcomes for intervention versus control patients, but which reported persistently high absolute rates of outcomes such as admissions and readmissions in each group.^48^ ^52^ ^54^ ^58^ ^65^ ^68^

This has implications for the potential success of policy initiatives designed to reduce hospital activity. In England, integration has become a central feature of the evolving healthcare policy landscape and there are high expectations of substantial benefits from integrating care. The BCF and ‘Vanguard’ sites^7^ have been developed following recognition that radically different models of care are needed if the NHS in England is to overcome its growing challenges, and both policy initiatives involve far-reaching change to health and social care services with the aim of meeting national headline targets for reduced hospital and emergency care use.^75^ Interventions shown to be effective in this review have much in common with the rationale behind the BCF—care provided in the community rather than in hospitals was shown in many cases to be highly effective. Multidisciplinary care, discharge planning and self-management educating patients on identifying symptoms of exacerbation of their condition(s) all have the potential to improve outcomes and reduce activity at the ‘back door’ and ‘front door’ of the acute sector. Disease-specific expertise was also found in many reviews to be crucial to the success of integrated care interventions, as was secondary care outreach to other settings. This bodes well for BCF and Vanguard initiatives built around these interventions. However, it is of concern that many vanguard sites aim to integrate care via a case management approach, which showed the poorest evidence of effectiveness in our review. This raises questions over whether the Vanguard strategies will be able to deliver the outcome improvements they are being established to achieve. The extent to which integrated care can bring about significant cost savings in a health system beset with ongoing budgetary constraints is also highly uncertain.

Interventions designed for single conditions were substantially more effective than those designed to treat patients with chronic diseases in general terms. On one hand, this suggests that service providers can achieve some ‘quick wins’ by targeting interventions such as discharge planning and specialist MDT towards specific patient groups in whom the evidence for reduced hospital use is clear. On the other hand, this means that integration may not deliver the substantial reductions in acute sector activity that must be achieved if healthcare services are to remain sustainable in the longer term.

### Unanswered questions and future research

Integrated care poses challenges to the measurement of ‘hard’ healthcare service outcomes in what are often complex intervention programmes. Determining cause and effect is difficult when interventions include multiple components, yet being able to link a specific intervention to a particular observed outcome is typically central to policymaking and commissioning objectives. Research to develop a robust taxonomy for integrated care interventions and their components would make assessments of comparative effectiveness across interventions less challenging. We attempted to maximise the relevance of review findings to the English health and social care system by considering interventions implemented in developed economies, but further research is needed to determine whether interventions found to be effective in other healthcare systems can be generalised to the NHS. In particular, robust evaluations would allow the influence of local and organisational contexts to be disentangled from the effects of the intervention themselves, as although the umbrella review gives some indication about ‘what’ might work, it does not necessarily help our understanding of ‘how’ an intervention works and why it may work in some circumstances and not others.

Few reviews explicitly addressed multimorbidity, which has recently become of central importance in debates about hospital use by patients with complex needs.^4^ ^76^ Further research is needed to understand the issues faced by patients with multimorbidity when negotiating the health and social care system.^77^ Similarly, despite our comprehensive search strategy, the evidence base focused little on the role of primary care, social care or the voluntary sector in providing integrated services. Given current policy drivers towards services being provided in the community by GPs and other organisations rather than acute providers, further research to assess the implications of integrated care for the organisation and delivery of services in these sectors is urgently needed.

## Conclusions

This review highlights a number of potentially effective integrated care interventions to reduce hospital use for patients with chronic diseases. Interventions based on MDT that include condition specialists, those focused on discharge management that include postdischarge rehabilitation and follow-up and those based on multicomponent strategies were most likely to be associated with significant reductions in hospital use for patients with single conditions such as heart failure and COPD. Yet there was little robust evidence about potential cost efficiencies, and the effectiveness of care delivered in primary and social care settings remains largely unknown. Despite considerable fanfare accompanying efforts to integrate care across the health and social care system in England, integration does not seem to be a ‘magic bullet’ and the magnitude of achievable gains is unlikely to match those required by current policy targets.
