# Supplementary material for: Does integrated care reduce hospital activity for patients with chronic diseases? An umbrella review of systematic reviews
Source: BMJ Open. 2016 Nov 21;6(11):e011952. doi: 10.1136/bmjopen-2016-011952 (PMC5129137; doi:10.1136/bmjopen-2016-011952)
Supplement: supplementary data [file bmjopen-2016-011952supp.pdf]

SUPPLEMENTARY INFORMATION

MEDLINE SEARCH STRATEGY

General chronic disease, multimorbidity and long term condition strategies

1 Chronic Disease/ (219572)
2 ((chronic$ or long-term) adj2 (diseas$ or ill$ or disorder$ or condition$)).ti,ab. (146254)
3 multiple morbidit$.ti,ab. (161)
4 multimorbidit$.ti,ab. (758)
5 or/1-4 (330552)
6 ((service or care) adj2 (provis$ or provide$ or pattern$ or delivery or access$ or model$)).ti,ab. (93810)
7 exp "Delivery of Health Care"/ (795692)
8 ((integrat$ or co-ordinat$ or multidisciplin$) adj2 (service$ or care or team$)).ti,ab. (17715)
9 ((case or self) adj2 management).ti,ab. (15942)
10 care plan$.ti,ab. (8063)
11 case finding.mp. or exp Case Management/ (11460)
12 (self manage$ or self-monitor$).ti,ab. (11658)
13 exp Self Care/ (40473)
14 telemedicine.mp. or exp Telemedicine/ (16316)
15 (patient$ adj (centre$ or center$ or centric or navig$ or liaison or advocat$)).ti,ab. (9019)
16 or/6-15 (901782)
17 5 and 16 (29837)
18 limit 17 to (english language and yr="2000 -Current" and "reviews (maximizes specificity)") (698)

Disease specific search terms

1 ((service or care) adj2 (provis$ or provision or pattern$ or access$ or deliver$ or model$)).ti,ab. (48530)
2 exp "Delivery of Health Care"/ (795692)
3 ((integrat$ or co-ordinat$ or multidisciplin$) adj2 (service$ or care or team$)).ti,ab. (17715)
4 ((case or self) adj2 management).ti,ab. (15942)
5 care plan$.ti,ab. (8063)
6 case finding.mp. or exp Case Management/ (11460)
7 (self manage$ or self-monitor$).ti,ab. (11658)
8 exp Self Care/ (40473)
9 telemedicine.mp. or exp Telemedicine/ (16316)
10 (patient$ adj (centre$ or center$ or centric or navig$ or liaison$ or advocate$)).ti,ab. (9015)
11 or/1-10 (876758)
12 exp Hypertension/ (210270)
13 exp Diabetes Mellitus/ (313614)
14 exp Cardiovascular Diseases/ (1871477)
15 Coronary Disease/ (126005)
16 exp Stroke/ (85417)
17 Ischemic Attack, Transient/ (17733)
18 exp Pulmonary Disease, Chronic Obstructive/ (36060)
19 exp Neoplasms/ (2557667)
20 cancer.mp. or exp Neoplasms/ (2691469)
21 Depression/ (76188)
22 exp Dementia/ (120330)
23 exp Arthritis/ (200553)
24 or/12-23 (5315877)
25 11 and 24 (159547)
26 limit 25 to (english language and yr="2000 -Current" and "reviews (maximizes specificity)") (2578)
